# Auditory perception dominates in motor rhythm reproduction

**DOI:** 10.1177/03010066221093604

**Published:** 2022-04-19

**Authors:** Alexandra Hildebrandt, Eric Grießbach, Rouwen Cañal-Bruland

**Affiliations:** Department for the Psychology of Human Movement and Sport, Institute of Sport Science, 9378 Friedrich Schiller University Jena, Germany

**Keywords:** multisensory integration, perception/action, temporal processing, modality appropriateness hypothesis, rhythm reproduction

## Abstract

It is commonly agreed that vision is more sensitive to spatial information, while audition is more sensitive to temporal information. When both visual and auditory information are available simultaneously, the modality appropriateness hypothesis predicts that, depending on the task, the most appropriate (i.e., reliable) modality dominates perception. While previous research mainly focused on *discrepant* information from different sensory inputs to scrutinize the modality appropriateness hypothesis, the current study aimed at investigating the modality appropriateness hypothesis when multimodal information was provided in a *nondiscrepant* and simultaneous manner. To this end, participants performed a temporal rhythm reproduction task for which the auditory modality is known to be the most appropriate. The experiment comprised an auditory (i.e., beeps), a visual (i.e., flashing dots), and an audiovisual condition (i.e., beeps and dots simultaneously). Moreover, constant as well as variable interstimulus intervals were implemented. Results revealed higher accuracy and lower variability in the auditory condition for both interstimulus interval types when compared to the visual condition. More importantly, there were no differences between the auditory and the audiovisual condition across both interstimulus interval types. This indicates that the auditory modality dominated multimodal perception in the task, whereas the visual modality was disregarded and hence did not add to reproduction performance.

## Introduction

Considering the variety of sensory inputs from the environment (Calvert et al., 1998), perception is by nature a multisensory process (Auvray & Spence, 2008; Calvert et al., 2004; Driver & Spence, 2000). For instance, when crossing a frequented street, pedestrians have to localize approaching vehicles by integrating available visual information (e.g., headlights) as well as auditory signals (e.g., horns or sirens) to generate a veridical and precise representation of the environmental circumstances (Spence, 2011) and to reduce perceptual ambiguity (Calvert et al., 1998). Certainly, this principle does not only apply to daily situations but also to more complex contexts involving time pressure, such as fast ball sports. For example, tennis players do not only rely on visual information from their opponents’ movements and ball flight to anticipate the ball's trajectory, but also derive information from the sound emanating from racquet-ball contact (e.g., Cañal-Bruland et al., 2018) or an opponent's grunt (e.g., Müller et al., 2019).

Given that different sensory inputs are processed with high spatial and temporal coincidence (cf. Bedford, 2001; Van Wassenhove et al., 2007), observers tend to attribute stimuli from different modalities to the same event resulting in the so-called *unity assumption* (cf. Jackson, 1953; Welch & Warren, 1980). However, an observer's assumption of unity does not necessarily imply that stimuli from different sensory sources contribute to perception to an equal extent. Bayesian approaches (see e.g., Körding et al., 2007; Körding & Wolpert, 2004), for instance, promote the fundamental idea that stimuli from different sensory modalities are weighted according to their informational value within a certain task. In addition, there are a plethora of studies suggesting that different sensory modalities interact and may even interfere with each other (for an overview, see Shimojo & Shams, 2001). In particular, there is evidence that the perceived intensity of a stimulus in one sensory modality is modulated by the simultaneous presentation of a second stimulus in another sensory modality (Sanabria et al., 2007; Shipley, 1964)—a phenomenon referred to as *intersensory bias* (Lukas et al., 2014; Welch & Warren, 1980). Following Welch and Warren (1980), the strength of intersensory bias is defined by structural factors (e.g., spatiotemporal discrepancy or coincidence) and cognitive factors (e.g., awareness on intersensory discrepancies, assumption of unity, compelling [i.e., stimulating] features of the situation).

Welch and Warren (1980) proposed that intersensory bias emerges because the perceptual system attempts to offer a percept that is most convenient for successfully solving the task at hand, implying that some modalities seem to be more suitable for certain task dimensions than others. In this regard, previous research predominantly focused on the visual modality (see e.g., Hutmacher, 2019) revealing an exceptionally robust bias of vision over audition, for instance, in terms of stimulus localization (Alais & Burr, 2004; Howard & Templeton, 1966; Lukas et al., 2014; Stratton, 1897) or speech perception (McGurk & MacDonald, 1976). According to Shimojo and Shams (2001), this strong effect supports the common assumption that human perception is first and foremost dominated by the visual modality. Despite this claim for the dominance of the visual modality, there is growing evidence that vision can also be dominated and altered by the auditory modality. Especially within the temporal domain, auditory stimuli were shown to dominate over visual stimuli in terms of judging interval duration and stimulus frequency (Burr et al., 2009; Gebhard & Mowbray, 1959; Recanzone, 2003; Shipley, 1964; Welch et al., 1986). Moreover, auditory information can also modify aspects of vision as sound signals have been shown to affect the perceived duration (Walker & Scott, 1981), stimulus intensity (Stein et al., 1996), and timing of a visual stimulus (Aschersleben & Bertelson, 2003; Fendrich & Corballis, 2001; Morein-Zamir et al., 2003; Parise & Spence, 2008; Shams et al., 2000) as well as manual interception (Tolentino-Castro et al., 2022). Additionally, auditory input can either increase or decrease visual temporal resolution (Shimojo et al., 2001) and alter the perceptual interpretation of an ambiguous (Sekuler et al., 1997) or nonambiguous visual event (Shams et al., 2000; Zampini & Spence, 2004).

By now, it is commonly agreed that the visual system has a higher resolution in spatial tasks whereas the auditory system is more sensitive in temporal tasks (Näätänen & Winkler, 1999; O’Connor & Hermelin, 1972; Recanzone, 2003, 2009; Sandhu & Dyson, 2012; Shimojo & Shams, 2001; Spence & Squire, 2003; Welch et al., 1986; Welch & Warren, 1980). A commonly proposed explanation for these modality-specific preferences is offered by the *modality appropriateness hypothesis (MAH)*, which is based on the notion that the sensory modalities, although each capable of various functions, are particularly specified to process information within appropriate dimensions (Freides, 1974; Lukas et al., 2014; O’Connor & Hermelin, 1972). In addition, the MAH is advocating the idea that the most appropriate (i.e., sensitive or reliable) modality will dominate perception within a multimodal task setting (Andersen et al., 2005; Matuz et al., 2019; Shimojo & Shams, 2001; Wada et al., 2003; Welch & Warren, 1980). According to Andersen et al. (2005) as well as Welch and Warren (1980), the appropriateness of a sensory modality is closely intertwined with attentional processes as human perception is proficient to estimate the relative reliability of different sensory sources and to purposefully direct attention toward the most reliable modality. The alignment of attention and, consequently, the processing of different sensory inputs due to the level of appropriateness are depending on stimulus characteristics (i.e., temporal, or spatial character, intensity, movement, salience, shape, size, orientation, texture; Shimojo & Shams, 2001; Welch & Warren, 1980) and task demands (e.g., whether it requires spatial or temporal processing; Lukas et al., 2014). Additionally, Welch and Warren (1980) reported that the more (temporally or spatially) complex a certain task, the more dominant the appropriate sensory modality will be.

While previous studies evaluating the premises of the MAH mainly used cross-modal switching tasks in which different sensory inputs provided *discrepant* information (see e.g., Lukas et al., 2010, 2014; Matuz et al., 2019; Sandhu & Dyson, 2012), however, it remains to be determined whether the *less* appropriate modality may or may not significantly add to successfully solving a task in a multimodal context for which (i) the most appropriate modality is known and (ii) all modalities provide *nondiscrepant* information. In other words: considering that different sensory inputs are not necessarily processed to the same extent although attributed to the same event (see e.g., Körding et al., 2007; Körding & Wolpert, 2004), and that task demands such as complexity seem to be of crucial importance to specify the *appropriateness* of sensory information from various modalities (see Welch & Warren, 1980), it is still an open question whether participants would benefit from additional and hence multimodal stimulation (as opposed to unimodal stimulation) if the task-dependent most appropriate modality was already addressed.

To examine this question and be able to compare unimodal versus multimodal processing (Welch & Warren, 1980), it is mandatory to first identify a task for which the most appropriate or reliable modality is known. Previous research, for instance, revealed a particularly distinguished bias toward the auditory modality for rhythm reproduction tasks in which participants were instructed to reproduce visual or auditory rhythmical patterns as temporally precisely as possible. With respect to the higher sensitivity of the auditory system to temporal information (cf., Loeffler et al., 2018; O’Connor & Hermelin, 1972; Recanzone, 2009; Sandhu & Dyson, 2012), this task has been identified to be favorably solved within the auditory modality as participants’ performance was significantly better when the rhythmical patterns were presented auditorily (cf., Chen et al., 2002; Gault & Goodfellow, 1938; Glenberg & Jona, 1991; Hove et al., 2013; Kolers & Brewster, 1985; Patel et al., 2005; Repp & Penel, 2004). For this reason, in the current study, we chose to modify the rhythm reproduction task which has been applied by Sarrazin et al. (2004, 2007) as we deemed their basic experimental setup suitable for our experimental endeavor.

Within a series of experiments, Sarrazin et al. (2004, 2007) provided participants with rhythmical sequences of visual or auditory origin, that is, either eight moving dots or eight sound beeps that simulated a moving object. Each (visual or auditory) pattern had to be reproduced from memory with spatial and temporal precision after a learning phase with either constant or variable interstimulus intervals (ISIs). Participants’ reproduction accuracy and variability were considered as dependent measures. Admittedly, Sarrazin et al. (2004, 2007) pursued different experimental goals by focusing on the unfolding effects of temporal information on spatial judgments (i.e., *tau effect*) as well as effects of spatial information on temporal judgments (i.e., *kappa effect*). Nonetheless, their stimulus configurations lend themselves to examine the research question outlined above, that is, whether participants would benefit from multimodal stimulation more than from unimodal stimulation. Thus, we designed an experiment in which participants were instructed to reproduce rhythmical patterns with different ISI configurations (i.e., constant or variable ISIs), which were either presented (i) auditorily (i.e., beeps), ii) visually (i.e., dots), or iii) audiovisually (i.e., simultaneous beeps and dots) to examine the impact of multimodal versus unimodal sensory inputs within a rhythm reproduction task and to further specify the assumptions of the MAH.

If it is true that a certain task is dominated by the most appropriate (i.e., most reliable) sensory modality or that certain tasks are more appropriate to be solved within a certain modality respectively (Freides, 1974; O’Connor & Hermelin, 1972; Welch & Warren, 1980), participants’ perception should be dominated by the auditory stimuli within our experimental setting. Consequently, as we chose a temporal precision task, we generally expected participants to perform better in the auditory than in the visual condition. In terms of the audiovisual condition, the MAH would predict that the most appropriate modality (i.e., here audition) attracts more attention than the less appropriate modality (i.e., here vision), resulting in a lower sensory impact of the visual modality for successful task solution (cf. Wada et al., 2003). According to Hass et al. (2012, p. 6), “in its most extreme form”, the MAH predicts that only the most appropriate modality might add to participants’ performance while the input from the less appropriate modality is fully neglected. If true, participants’ accuracy and variability should not differ between the auditory and the audiovisual condition. Additionally, bearing in mind that an increasing temporal task complexity might lead to a more pronounced effect of modality appropriateness (cf. Welch & Warren, 1980), the difference between the auditory (or even audiovisual) and the visual condition is predicted to be larger in variable than in constant ISI configurations.

## Method

### Participants

Based on an estimated effect size of *η_p_*² = .20, which is consistent with similar studies (e.g., Glenberg & Jona, 1991), a power analysis conducted in GPower (Version 3.1) resulted in a sample size of 34 participants. Considering the possibility of participant drop out, we recruited 40 participants (*M*_age_ = 25.7 years, *SD*_age_ = 3.9 years; 15 male, 25 female) who volunteered to take part in the experiment. All participants had normal or corrected-to-normal hearing as well as vision (both based on self-report) and provided informed consent prior to experimentation. The study design was approved by the ethics committee of the Faculty of Social and Behavioral Sciences of Friedrich Schiller University Jena (FSV 21/026).

### Apparatus

The experiment was conducted on a desktop computer (Fujitsu Celsius M740, Fujitsu Technology Solutions GmbH, Tokyo, Japan) using a 24″ screen with a refreshing rate of 60 Hz (Fujitsu P24W-7, Fujitsu Technology Solutions GmbH, Tokyo, Japan) and a wired keyboard (Fujitsu KBPC PX ECO, Fujitsu Technology Solutions GmbH, Tokyo, Japan). For the presentation of the auditory stimuli, we used over-ear headphones (Sony MDR-ZX110, Sony Corporation, Tokyo, Japan). The experiment was created using the PsychoPy3 interface (Version 2021.1.4.; cf. Peirce et al., 2019; see https://osf.io/ycf2s/?view_only=f0117e75e44c49adafa448c4eb872630).

### Stimuli

**Figure 1.**
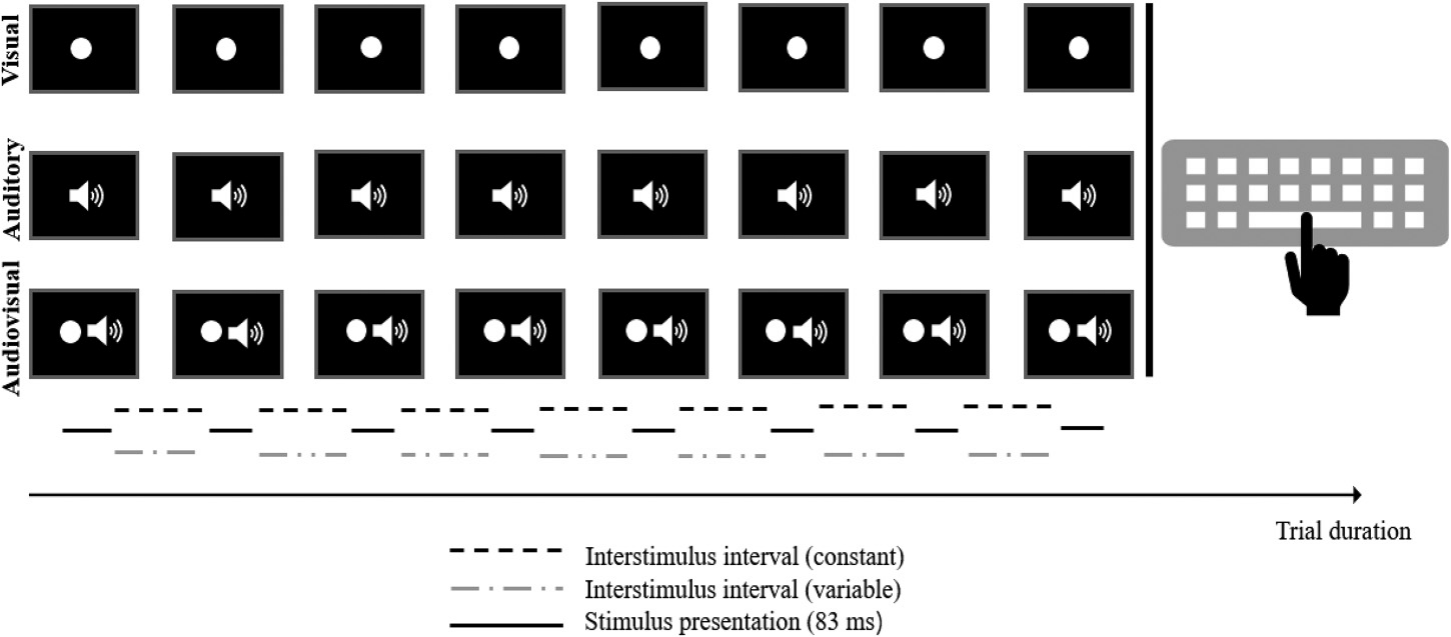
Schematic illustration of the stimulus setup and material for the three experimental conditions.

The current experiment comprised three conditions with different stimuli setups (see Figure 1). Within each condition, eight stimuli were presented sequentially, thereby generating a rhythmical pattern. The design of our stimulus material (e.g., number of stimuli per pattern, variations in terms of ISI, stimulus appearance) was based on Sarrazin et al. (2004, 2007). With respect to our experimental purpose, however, we made some necessary adjustments: For the visual condition, a flashing white circle with a diameter of 9.6 cm was presented in the center of the screen. For the auditory condition, we used a sound with a frequency of 440 Hz. Within the audiovisual condition, the visual and auditory stimuli were presented simultaneously. Independent of condition, each stimulus was presented for 83 ms (i.e., stimulus duration of five frames). To implement different ISI types (cf. Sarrazin et al., 2004, 2007), the experimental stimuli were either shown with constant (i.e., equal intervals between stimuli) or variable (i.e., different intervals between stimuli) ISIs. In general, ISIs were defined as the intervals between the offset of one stimulus and the onset of the next stimulus. Similar to the experiments by Sarrazin et al. (2004, 2007), the ISIs varied between 278 and 795 ms (i.e., 12–43 frames). As far as possible, the duration of variable ISI combinations was matched to the duration of constant ISI combinations except for the shortest (278 ms) and longest (795 ms) ISIs as no other combination was capable to create the same duration. In sum, this resulted in 32 constant and 32 variable ISI configurations, which were included in all three experimental conditions. A more detailed illustration of the ISI setup can be found in the Supplemental Material.

### Procedure

In advance of the experiment, participants were briefed about the experimental procedure. That is, they were informed about the experimental modalities (i.e., the blocked design with visual, auditory and audiovisual stimulus configurations) and the number of stimuli to reproduce for each rhythmical pattern. Participants were instructed to reproduce the given rhythmical patterns via key press (space bar) on the keyboard as temporally precisely as possible. The experimental instructions were presented on the computer screen so that the participants could control the course of the experiment on their own. The experiment comprised three experimental blocks, each of which represented one of the three experimental conditions (i.e., audiovisual vs. auditory vs. visual). Participants passed through all three blocks in counterbalanced order, yielding a classical within-subject design. There were 64 randomized trials in each block, 32 with constant and 32 with variable ISI structure. Each block started with 10 practice trials in which participants received feedback about their performance (i.e., information regarding their average temporal deviation and if they were too early or too late) to get familiar with the stimulus material. Next, they started with the experimental trials in which no feedback was provided. In between blocks, participants were given the opportunity to take a short break. In total, the experiment included 192 experimental trials and took ∼60 min to complete.

### Data Analysis

The temporal deviation between the presented and the reproduced ISI (i.e., the interval between two consecutive key presses) was considered our dependent measure. In particular, we calculated the constant error (CE) and the variable error (VE) in line with Welch and Warren (1980) as well as Sarrazin et al. (2004, 2007). The CE marks the difference between participants’ response time for two successive key presses (i.e., RT) and the sum of the presented ISI (i.e., provided within the rhythmical pattern; $\mathrm{IS}I_{p}$ ) and the stimulus duration of 83 ms:

$$\mathrm{CE}=\mathrm{RT}-(\mathrm{IS}I_{p}+83\;\mathrm{ms}).$$

It defines participants’ reproduction accuracy and determines whether participants are biased to press the space bar too late or too early. The VE describes the absolute difference between the mean CE of a certain condition ( $\bar{x}$ ) and the CE of each response:

$$\mathrm{VE}=|\bar{x}-\mathrm{CE}|.$$

It defines the deviation of the CE from the level-specific mean (i.e., specific to subject, condition, and ISI structure). Consequently, the VE is a measure of response-to-response variability for the reproduced ISIs without the temporal bias (cf. Schutz & Roy, 1973).

Data analyses were conducted using R (Version 4.1.2, R Foundation, Vienna, Austria). To examine whether the dependent measures were affected by condition and/or ISI type according to our hypotheses, two separate 3 (condition: auditory vs. audiovisual vs. visual) by 2 (ISI type: constant vs. variable) analyses of variance (ANOVAs) were run for the CE (reproduction accuracy) and the VE (reproduction variability), respectively. Additionally, we conducted post-hoc pairwise comparisons with Bonferroni–Holm correction to specify the results of the ANOVAs. The effect sizes for analyses of variance are reported as partial eta squared (η*_p_*^2^). For post-hoc pairwise comparisons, we report β as an indicator for the mean difference with the corresponding 95% confidence intervals as well as Cohen's *d* as effect size. Alpha was set at 0.05 for all statistical analyses.

## Results^1^

### CE—Reproduction Accuracy

**Figure 2.**
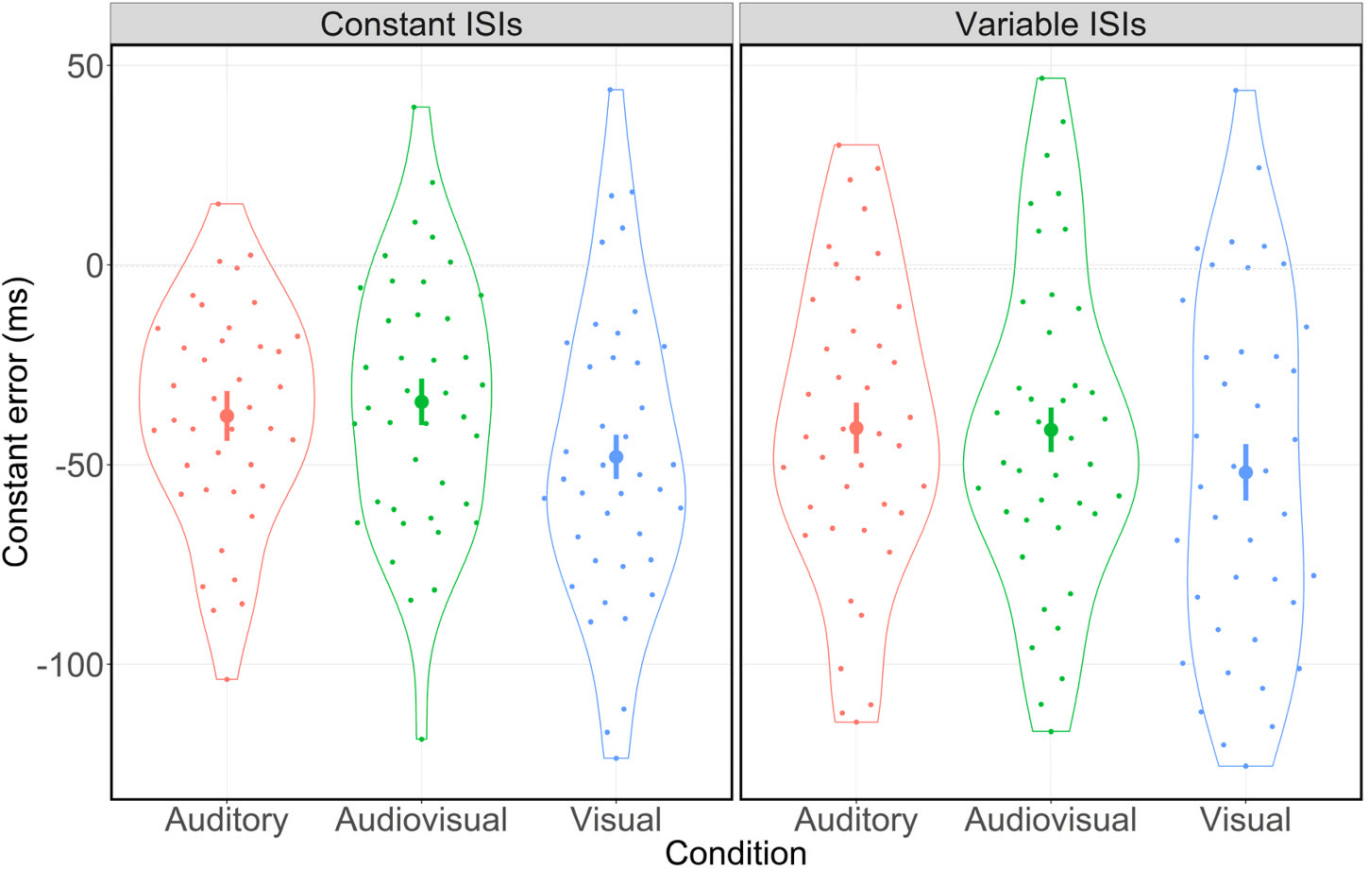
Distribution of the CE in ms for constant and variable ISIs separated by condition. Error bars indicate 95% confidence intervals. Dots represent mean values for each participant. Jitters are for clarification purposes only.

*Note*. CE=constant error; ISI=interstimulus interval.

As illustrated in Figure 2, participants’ rhythm reproduction appeared to be more accurate in the presence of auditory input. Specifically, the 3 (condition: auditory vs. audiovisual vs. visual) by 2 (ISI type: constant vs. variable) ANOVA for the CE revealed a significant main effect for condition (*F*(2,78) = 6.14, *p* = .003, *η_p_*² = 0.14). As there was neither a significant main effect for ISI type (*F*(1,39) = 2.51, *p* = .122, *η_p_*² = 0.06) nor an interaction effect between condition and ISI type (*F*(2,78) = 0.99, *p* = .375, *η_p_*² = 0.03), these results indicate that participants’ reproduction accuracy was affected by condition only. That is, participants’ CEs differed significantly between the three conditions. Post-hoc pairwise comparisons showed significant differences with respect to participants’ reproduction accuracy between the auditory and the visual (*β* = −10.70 ms, 95% CI [−19.05, −2.36], *Cohen's d* = 0.41, *p* = .013) as well as the audiovisual and the visual (*β* = −12.23 ms, 95% CI [−20.02, −4.45], *Cohen's d* = 0.50, *p* = .003) condition. However, there was no significant difference between the auditory and the audiovisual condition (*β* = 1.53 ms, 95% CI [−5.35, 8.41], *Cohen's d* = 0.07, *p* = .656). In sum, participants were significantly more accurate in the auditory and the audiovisual condition. Additionally, participants generally displayed a significant bias toward an early action (see also Figure 2). That is, they tended to press the space bar too early independent of ISI structure (one-sampled *t*-test: *x̄* = −42.35 ms, 95% CI [−52.74, −31.97], *Cohen's d* = 1.30, *p* < .001).

### VE—Reproduction Variability

**Figure 3.**
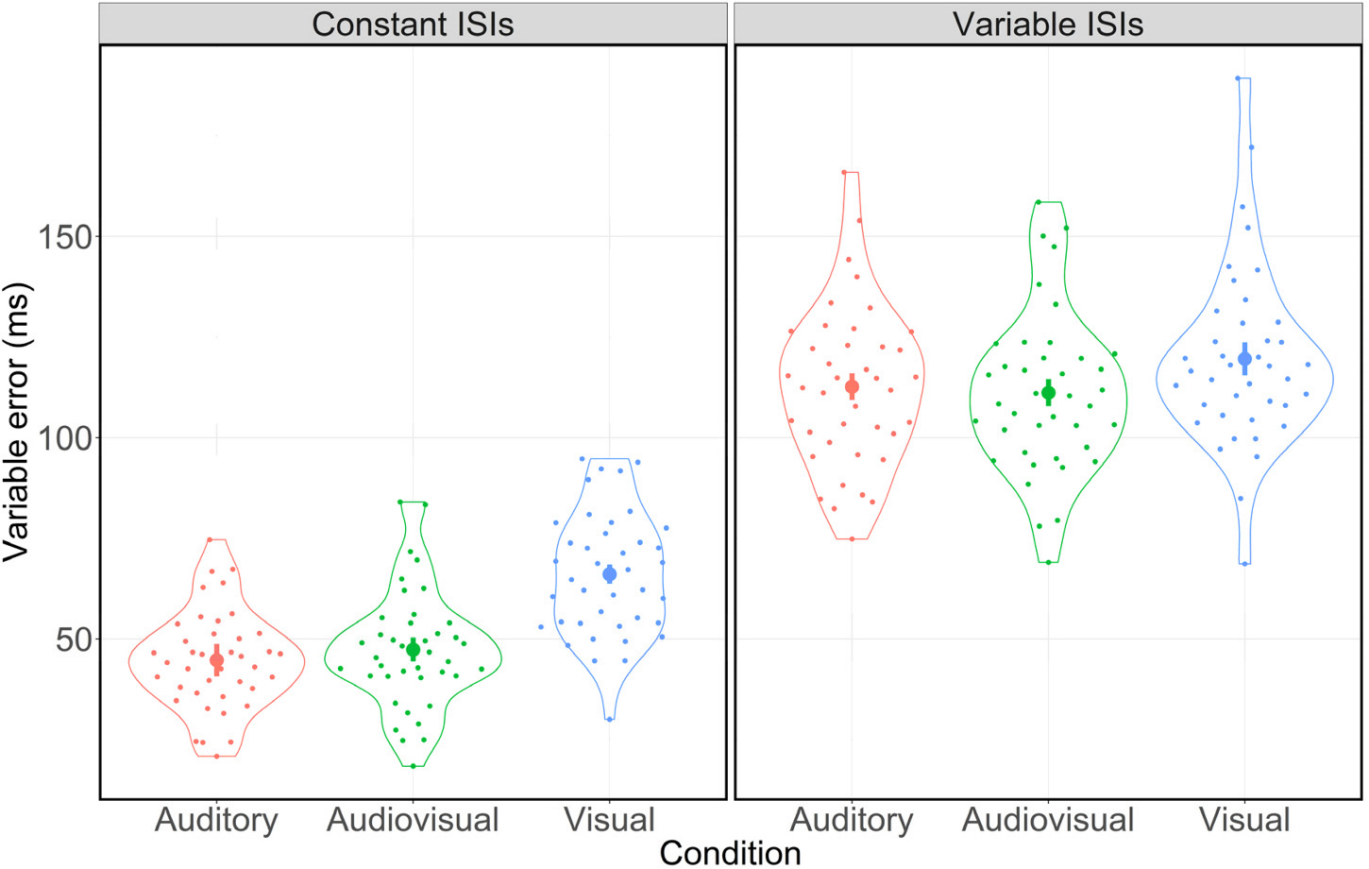
Distribution of the VE in ms for constant and variable ISIs separated by condition. Error bars indicate 95% confidence intervals. Dots represent the mean values for each participant. Jitters are for clarification purposes only.

*Note*. VE=variable error; ISI=interstimulus interval.

As shown in Figure 3, participants’ rhythm reproduction appeared to be less variable in the presence of auditory input. Indeed, the 3 (condition: auditory vs. audiovisual vs. visual) by 2 (ISI type: constant vs. variable) ANOVA for the VE revealed a significant main effect for condition (*F*(2,78) = 48.39, *p* < .001, *η_p_*² = 0.55) and for ISI type (*F*(1,39) = 945.67, *p* < .001, *η_p_*² = 0.96), indicating that participants’ VEs differed significantly between the three conditions and between both ISI types. Additionally, there was a significant interaction between condition and ISI type (*F*(2,78) = 18.84, *p* < .001, *η_p_*² = 0.33), revealing that the manifestation of variability differences between the three conditions was affected by ISI type.

For constant ISIs, post-hoc pairwise comparisons showed significant differences between the auditory and the visual condition (*β* = 21.35 ms, 95% CI [17.39, 25.32], *Cohen's d* = 1.73, *p* < .001) as well as between the audiovisual and the visual condition (*β* = 18.75 ms, 95% CI [15.19, 22.30], *Cohen's d* = 1.69, *p* < .001). Again, there were no significant differences between the auditory and the audiovisual condition (*β* = 2.61 ms, 95% CI [−0.82, 6.03], *Cohen's d* = 0.24, *p* = .131).

For variable ISIs, post-hoc pairwise comparisons also showed significant differences between the auditory and the visual condition (*β* = 6.92 ms, 95% CI [−2.10, 11.74], *Cohen's d* = 0.46, *p* = .012) as well as between the audiovisual and the visual condition (*β* = 8.39 ms, 95% CI [3.46, 13.32], *Cohen's d* = 0.54, *p* = .004). There were no differences between the auditory and the audiovisual condition (*β* = −1.47 ms, 95% CI [−5.14, 2.20], *Cohen's d* = 0.13, *p* = .422). That is, participants’ VE was smaller in the presence of auditory input. However, this effect was attenuated for variable ISIs.

In terms of ISI type, post-hoc pairwise comparisons revealed significant differences between the VEs for constant and variable ISIs within the auditory condition (*β* = 67.92 ms, 95% CI [62.24, 73.60], *Cohen's d* = 3.82, *p* < .001), the audiovisual condition (*β* = 63.84 ms, 95% CI [59.19, 68.51], *Cohen's d* = 4.38, *p* < .001) and the visual condition (*β* = 53.49 ms, 95% CI [49.07, 57.90], *Cohen's d* = 3.87, *p* < .001). These results indicate a significant increase of participants’ reproduction variability for variable ISIs in all conditions.^2^

## Discussion

According to the MAH, when solving a task for which different sensory channels provide input, the most appropriate (i.e., sensitive or reliable) modality will dominate perception (Hass et al., 2012; Lukas et al. 2014; Welch & Warren, 1980). The current study aimed at scrutinizing the premises of the MAH in a multimodal setting by comparing the effects of nondiscrepant multimodal (audiovisual) versus unimodal (auditory & visual) stimulation in a rhythm reproduction task, which had previously been identified to be favorably solved within the auditory modality (cf. Chen et al., 2002; Hove et al., 2013; Patel et al., 2005; Repp & Penel, 2004). Besides controlling for modality appropriateness, we manipulated task complexity by administering different ISIs (i.e., constant and variable; cf. Sarrazin et al., 2004, 2007) to further examine whether the effect of modality appropriateness would be more pronounced in more complex tasks, that is, the variable ISI conditions as opposed to the constant ISI conditions (Welch & Warren, 1980).

Results mainly confirmed our predictions with respect to the MAH. First, participants were significantly more accurate and less variable in the auditory condition than in the visual condition across both ISI types indicating that our paradigm reliably induced effects of modality appropriateness in favor of the auditory modality. Second, and addressing the main research question whether in a multimodal stimulus environment, an additionally available but *less* appropriate modality may or may not add to solving the task, there were no significant differences between the auditory (unimodal) and the audiovisual (multimodal) condition with respect to both dependent measures and ISI types. If, as discussed by Andersen et al. (2005) as well as Welch and Warren (1980), the appropriateness of a sensory modality is closely related to directing attention toward the most reliable modality, our results might indicate that attentional resources in the audiovisual condition were (solely) focused on the auditory stimuli while the visual stimuli were disregarded (cf. Chen et al., 2002; Hass et al., 2012; Repp & Penel, 2004; Wada et al., 2003; Welch & Warren, 1980).

Additionally, our findings might be in line with Lukas et al. (2014) who claim that temporal tasks would always be dominated by auditory input—even if different sensory inputs are available. In keeping with Matuz et al. (2019), this dominance effect results from processing differences between auditory and visual stimuli in temporal tasks. That is, visual stimuli transport less accurate temporal information and also require more cognitive resources to be processed which is why participants’ pattern reproductions within the audiovisual condition might have been essentially and primarily guided by auditory stimuli (cf. Repp & Penel, 2004). Interestingly, our participants subjectively confirmed this assumption reporting in an exit interview after the experiment that they had mainly focused on the auditory input in the audiovisual condition.

As introduced before, Welch and Warren (1980) hypothesized that a more complex task (i.e., in terms of spatial or temporal demands) would result in a more pronounced effect of modality appropriateness. In line with Sarrazin et al. (2004, 2007), we therefore manipulated temporal task complexity by implementing constant as well as variable ISIs. Our results do not support the original assumption. Although our results generally revealed more accurate and less variable performances for the auditory (and the audiovisual) condition across both constant and variable ISIs, the effects were smaller as concerns performance variability in variable ISI conditions than in constant ISI conditions. That is, VEs were (i) significantly larger across all conditions with variable ISIs when compared to constant ISIs and (ii) the differences between the auditory and the visual as well as between the audiovisual and the visual condition diminished. One methodological explanation for this finding might be that our ISI manipulations (i.e., variable ISIs) may not only have increased *temporal* task complexity, but rather *general* task complexity. Supposing that a more pronounced effect of appropriateness would manifest itself by an increased difference in variance between the auditory and the visual condition, it might even be possible that our ISI manipulations caused the opposite effect as the appropriateness of the task might have actually decreased. If true, the smaller differences between conditions might indicate that the nondominant (i.e., less appropriate) visual modality which had no additional effect on perception within constant ISIs increasingly contributed to participants’ performance to overcome perceptual uncertainty within variable ISIs (Welch & Warren, 1980). Regardless, the modality appropriateness effect in favor of the auditory modality proved robust independent of ISI type.

Next to the modality appropriateness effect, results revealed a bias towards acting early, as demonstrated by a consistent shift in the CE, indicating that participants’ key presses were consistently too early. This tendency seems to be in line with the so-called negative asynchrony as introduced by Repp (2005). In his review, Repp (2005) highlighted that in tapping tasks participants’ taps generally tend to precede the external rhythm (see also Yang et al., 2019). However, with respect to our results, this early bias was significantly more pronounced in the visual condition. In this regard, Jäncke et al. (2000) suggest that auditory stimuli generate an internal rhythm (i.e., a kind of internal pacemaker) whereas visual stimuli do not or less so due to their lower temporal resolution. Assuming that this internal rhythm crucially assists a temporally precise rhythm reproduction as it might lead to a more robust and durable internal representation of the rhythmical patterns (Chen & Spence, 2017; Holcombe, 2009), one might speculate that the earlier responses in the visual condition might corroborate the attempt of the visual system to compensate for the deficit in generating an internal rhythm.

In the remainder of the discussion, we would like to address further directions for future research on the interaction of sensory modalities. First, although our data indicate a dominance effect of the most appropriate modality for task solution, current approaches such as Bayesian integration models (see e.g., Colombo & Seriès, 2012; Körding et al., 2007; Körding & Wolpert, 2004; Turner et al., 2017) clearly advocate a weighting hypothesis according to which the VE would be expected to be lowest under multimodal conditions due to the highest informational value and the statistically optimal integration of multiple sources of information respectively (Alais & Burr, 2004; Ernst & Bülthoff, 2004; Körding & Wolpert, 2004). Interestingly, an initial, preliminary Bayesian analysis based on our data (for details, see Supplemental material), does not confirm this assumption as the corresponding estimate for audiovisual integration differed significantly from the actual standard deviation within the audiovisual condition. Although further research and analyses are certainly needed, our exploratory analysis also supported the modality appropriateness effect in favor of the auditory modality.

Second, as already stated by Lukas et al. (2014), future research would be well-advised to further scrutinize the effects of (temporal) task complexity on modality appropriateness not only in terms of general task properties (Gil & Droit-Volet, 2011), but also with respect to other factors such as stimulus location (Kliegl & Huckauf, 2014), the presence of a second task (Brown, 2008), the attention aligned to the stimulus (Macar et al., 1994; Tse et al., 2004), affective states (Angrilli et al., 1997) or temporal coincidence between auditory and visual stimuli (Jones & Jarick, 2006). As already suggested by Sarrazin et al. (2004, 2007), it would also be noteworthy to examine (interindividual) differences in the manifestation of modality appropriateness effects. This is particularly interesting with respect to rhythm reproduction ability and memory capabilities as some studies already introduced, for instance, age effects in temporal estimation (Espinosa-Fernández et al., 2003) as well as gender differences in memory recall (Baer et al., 2006).

To conclude, the current study provided evidence for the MAH in a rhythm reproduction task. That is, rhythm reproduction was most accurate and precise when the most appropriate modality “audition” was available. In addition, when audiovisual information was available, the additional presence of less appropriate visual information did not add to rhythm reproduction but was instead discarded.

## Supplemental Material

sj-docx-1-pec-10.1177_03010066221093604 - Supplemental material for Auditory perception dominates in motor rhythm reproduction Click here for additional data file. Supplemental material, sj-docx-1-pec-10.1177_03010066221093604 for Auditory perception dominates in motor rhythm reproduction by Alexandra Hildebrandt, Eric Grießbach and Rouwen Cañal-Bruland in Perception

## Notes

1. Following the suggestion of an anonymous reviewer, we also calculated a Pearson's product moment corre-lation between the CE and the VE to investigate the relationship between our dependent measures. The analysis revealed a negative but nonsignificant correlation (*r* = −0.11, *p* = .48, 95% CI [−0.41, 0.20]).

2. Again, following the suggestion of an anonymous reviewer, we conducted two additional 3 (condition: auditory vs. audiovisual vs. visual) by 2 (ISI type: constant vs. variable) by 2 (time: first 16 trials vs. last 16 trials) ANOVAs to examine whether the magnitude and direction of the observed differences were the same at the beginning versus the end of the experiment. Results revealed that there were no significant main effects for time, no significant two-way interactions (between time and condition or time and ISI type), and no significant three-way interactions (all *p* > .19). This was true for both the CE and the VE.
