# Supplementary material for: Auditory perception dominates in motor rhythm reproduction
Source: Perception. 2022 Apr 19;51(6):403–16. doi: 10.1177/03010066221093604 (PMC9121532; doi:10.1177/03010066221093604)
Supplement: sj-docx-1-pec-10.1177_03010066221093604 - Supplemental material for Auditory perception dominates in motor rhythm reproduction [file sj-docx-1-pec-10.1177_03010066221093604.docx]

**Auditory perception dominates in motor rhythm reproduction**

Alexandra Hildebrandt, Eric Grießbach & Rouwen Cañal-Bruland

Department for the Psychology of Human Movement and Sport, Institute of Sport Science, Friedrich Schiller University Jena, Germany

***Corresponding author:***

Alexandra Hildebrandt, Institute of Sport Science, Friedrich Schiller University Jena, Seidelstraße 20, 07749 Jena, Germany; Email: alexandra.katrina.hildebrandt@uni-jena.de

**Supplementary material**

**Detailed setup of ISI configurations**

Within the main article, we briefly introduced the use of 32 constant as well as 32 variable ISI configurations which were matched according to their duration. For the sake of transparency, detailed data for each ISI are provided in Table S1 (for constant ISIs) and Table S2 (for variable ISIs).

**Table S1. Constant ISI configurations.**  Listed below, the 32 constant ISI configurations are illustrated in more detail including the respective ISI sequence as well as the total trial duration (i.e., the sum of the seven ISIs for each trial).

| ISI configuration | ISI (in ms) sequence | Total trial duration in ms |
| --- | --- | --- |
| 1 | 278 278 278 278 278 278 278 | 1946 |
| 2 | 295 295 295 295 295 295 295 | 2065 |
| 3 | 311 311  311  311  311  311  311 | 2177 |
| 4 | 328 328  328  328  328  328  328 | 2296 |
| 5 | 345  345  345  345  345  345  345 | 2415 |
| 6 | 361  361  361  361  361  361  361 | 2527 |
| 7 | 378  378  378  378  378  378  378 | 2646 |
| 8 | 395  395  395  395  395  395  395 | 2765 |
| 9 | 411  411  411  411  411  411  411 | 2877 |
| 10 | 428  428  428  428  428  428  428 | 2996 |
| 11 | 445  445  445  445  445  445  445 | 3115 |
| 12 | 461  461  461  461  461  461  461 | 3227 |
| 13 | 478  478  478  478  478  478  478 | 3346 |
| 14 | 495  495  495  495  495  495  495 | 3465 |
| 15 | 511  511  511  511  511  511  511 | 3577 |
| 16 | 528  528  528  528  528  528  528 | 3696 |
| 17 | 545  545  545  545  545  545  545 | 3815 |
| 18 | 561  561  561  561  561  561  561 | 3927 |
| 19 | 578  578  578  578  578  578  578 | 4046 |
| 20 | 595  595  595  595  595  595  595 | 4165 |
| 21 | 611  611  611  611  611  611  611 | 4277 |
| 22 | 628  628  628  628  628  628  628 | 4396 |
| 23 | 645  645  645  645  645  645  645 | 4515 |
| 24 | 661  661  661  661  661  661  661 | 4627 |
| 25 | 678  678  678  678  678  678  678 | 4746 |
| 26 | 695  695  695  695  695  695  695 | 4865 |
| 27 | 711  711  711  711  711  711  711 | 4977 |
| 28 | 728  728  728  728  728  728  728 | 5096 |
| 29 | 745  745  745  745  745  745  745 | 5215 |
| 30 | 761  761  761  761  761  761  761 | 5327 |
| 31 | 778  778  778  778  778  778  778 | 5446 |
| 32 | 795  795  795  795  795  795  795 | 5565 |

**Table S2. Variable ISI configurations.**  Listed below, the 32 variable ISI configurations are illustrated in more detail including the respective ISI sequence as well as the total trial duration (i.e., the sum of the seven ISIs for each trial).

| ISI configuration | ISI (in ms) sequence | Total trial duration in ms |
| --- | --- | --- |
| 1 | 311 278 278 295 311 278 311 | 2062 |
| 2 | 295  278  278  278  311  311  311 | 2062 |
| 3 | 278  278  295  311  461  278  278 | 2179 |
| 4 | 278  361  428  278  295  361  295 | 2296 |
| 5 | 411  345  278  395  311  295  378 | 2413 |
| 6 | 311  461  328  395  378  278  378 | 2529 |
| 7 | 295  311  345  311  278  361  745 | 2646 |
| 8 | 311  345  278  361  545  528  295 | 2763 |
| 9 | 378  428  295  395  478  428  478 | 2880 |
| 10 | 545  345  411  578  295  545  278 | 2997 |
| 11 | 795  528  395  295  478  311  311 | 3113 |
| 12 | 395  278  328  611  661  628  328 | 3229 |
| 13 | 495  778  328  311  428  295  711 | 3346 |
| 14 | 511  478  645  411  445  295  678 | 3463 |
| 15 | 611  545  278  761  361  328  695 | 3579 |
| 16 | 461  511  478  561  278  678  728 | 3695 |
| 17 | 478  745  511  328  645  695  411 | 3813 |
| 18 | 428  678  345  395  745  578  761 | 3930 |
| 19 | 461  761  711  728  711  278  395 | 4045 |
| 20 | 711  711  511  445  678  511  595 | 4162 |
| 21 | 478  445  778  478  795  511  795 | 4280 |
| 22 | 795  728  361  661  611  778  461 | 4395 |
| 23 | 295  778  778  628  728  661  645 | 4513 |
| 24 | 778  578  728  795  645  761  345 | 4630 |
| 25 | 728  511  661  545  795  795  711 | 4746 |
| 26 | 295  778  745  761  761  728  795 | 4863 |
| 27 | 661  795  778  628  661  711  745 | 4979 |
| 28 | 645  795  728  711  761  761  695 | 5096 |
| 29 | 795  628  778  795  711  761  745 | 5213 |
| 30 | 795  728  761  795  745  778  728 | 5330 |
| 31 | 795  795  795  728  778  761  795 | 5447 |
| 32 | 778  711  795  795  778  795  795 | 5447 |

**Multimodal Bayesian estimation of variance**

In the discussion section of the main article, we referred to a Bayesian estimation of variance as an alternative model for the perception of multimodal inputs. To investigate whether participants exerted Bayesian inference, we compared the standard deviations from a Bayesian estimator with our empirical data. According to the Bayesian equation, the combination of information from multiple sensory sources (here auditory and visual) improves the precision of the estimate. A Bayesian posterior variance ($\sigma_{Post}^{2}$) of the audiovisual condition is given by the following equation (assuming normal distributions of the posterior distributions under all conditions):

$$\sigma_{Post(i)}^{2}=\frac{1}{\frac{1}{\sigma_{vis(i)}^{2}}+\frac{1}{\sigma_{aud(i)}^{2}}} (1)$$

, where $\sigma_{vis(i)}^{2}$ is the variance of response time from the visual conditions and $\sigma_{aud(i)}^{2}$is the variance of the response time for the auditory condition for every individual (i) separately.

Figure S1 displays the standard deviation $(\sqrt{\sigma})$ for the Bayesian estimation compared to the other conditions. The standard deviation for the audiovisual Bayes estimation was smaller than the empirical audiovisual standard deviation, especially in terms of variable ISIs. This difference is also illustrated by the significant interaction from the 2 (condition: audiovisual (Bayes) vs. audiovisual) by 2 (ISI type: constant vs. variable) ANOVA (see Table S3) and the following post-hoc pairwise comparison between the audiovisual vs. audiovisual (Bayes) condition for each ISI type (see Table S4). These results indicate that participants did not perform Bayes optimal multimodal estimations.





**Fig S1. Distribution of the standard deviation in ms for each condition separated by ISI type.** As illustrated, the Bayesian estimate for audiovisual integration would predict the lowest standard deviation for the combination of both sensory sources due to the assumption of highest informational value. However, this was not the case for the actual standard deviation of the audiovisual condition in our experimental setting.

**Table S3. ANOVA statistics.**  The 2 (condition: audiovisual (Bayes) vs. audiovisual) by 2 (ISI type: constant vs. variable) ANOVA for standard deviation revealed significant main effects for condition and ISI type as well as a significant interaction between condition and ISI type.

| Effect | F (1, 39) | p | η_p_² |
| --- | --- | --- | --- |
| Condition | 187.12 | <0.05 | 0.828 |
| ISI type | 640.99 | <0.05 | 0.943 |
| Condition x ISI type | 35.493 | <0.05 | 0.476 |

**Table S4. Post-hoc pairwise comparison for the audiovisual (Bayes) vs. the audiovisual condition.** With respect to the significant 2x2 interaction, post-hoc pairwise comparisons between the Bayesian and the empirical standard deviation for the audiovisual condition were conducted for both ISI types.

| ISI type | Group 1 | Group 2 | t (39) | Diff (ms) | 95% CI (ms) | p |
| --- | --- | --- | --- | --- | --- | --- |
| constant | audiovisual  (Bayes) | audiovisual | -6.45 | -19.71 | [-25.90 to -13.5] | <0.05 |
| variable | audiovisual  (Bayes) | audiovisual | -14.87 | -42.27 | [-48.02 to -36.52] | <0.05 |
